# The Relative Efficiency of Various Anæsthetics

**Published:** 1898-07

**Authors:** 


### Abstracts and Translations.

### THE RELATIVE EFFICIENCY OF VARIOUS ANÆSTHETICS.

THE *British Medical Journal* of November 20, 1897, speaks editorially of Waller's paper on anæsthetics,[1] in which he discusses the relative advantages of ether and chloroform. Dr. Waller's experiments have convinced him that the most satisfactory way of testing the efficiency of anæsthetics is to employ freshly prepared nerves, and to cause them to respond to electrical stimuli while subjected to various narcotizing vapors. The method is as beautiful as it is original. Not only is he enabled thus to investigate the anæsthesia produced, or, as he terms it, "temporary immobilization," but to ascertain the strength of vapor which kills the nerve and produces "permanent immobilization." Under ordinary physi-

[1] Published in the British Medical Journal of same date.

ological conditions the galvanometer reveals the presence of currents in the nerves. The action of narcotics is to send the nerve to sleep, and the absence of the current is proof positive of this anæsthetic sleep. Remove the vapor and the nerve recovers itself, and again the nerve currents appear. Now, by testing a number of substances, Dr. Waller has found that while all those which produce anæsthesia also bring about death of the nerve, some are far more deadly than others. Richardson, in his lectures on narcotic vapors, was disposed to regard the chemical and physical properties of narcotics as affording no criteria of their power of anæsthetics, but his methods lacked the precision of those now under consideration. The heart is capricious because it is a compound organ with a nerve factor, a muscular factor, and a nerve-cell factor. Dealing with isolated nerve the problem becomes immeasurably simpler, and at the same time the experiments can be kept absolutely under test conditions.

We have hitherto been assured that chloroform killed either by paralyzing respiration through the nervous system, leaving the other tissues severely alone, or brought about death through circulatory failure, due, it may be, to vasomotor dilatation, or through incapacitating the heart. Now we learn that not only does chloroform destroy muscular tissue and narcotize the nervous centres through its influence on the blood, but actually attacks nerve fibrillæ, and destroys their irritability. It is a matter of indifference to "the man in the street," as Dr. Waller points out, whether he is killed through his heart failing or by asphyxia; what interests him most is how he can be saved pain with as little chance of losing his life as his surgeon can conveniently arrange. From this point of view it is somewhat startling to find that dose for dose chloroform is seven times more deadly than ether when tried upon the isolated nerve,—that is, upon a highly specialized protoplasm. And what appeals most to the practical man is that these figures show how narrow a margin in the case of chloroform there is between a non-lethal and a lethal dose, while seven times that margin exists when ether is in use. The specific action of nitrous oxide has been asserted by some, but denied by Wood and Cerna and the late Sir George Johnson. Dr. Kemp's careful study confirms the former view, but according to Dr. Waller it is an anæsthetic which has little or no action on nervous tissue. Carbonic dioxide, which Snow and Richardson lauded as an anæsthetic, is, Dr. Waller finds, a powerful agent to produce "immobilization," thus confirming the assumption of the older observers. But a fact of extreme interest, because unless carefully explained liable to fatal misunderstanding, is Dr. Waller's observation that the presence of carbon dioxide favors anæsthesia and lessens its risk. The combination of chloroform and carbonic oxide is not, it must be distinctly understood, simply chloroform given with very little air. This distinction is made very plain by Dr. Waller himself.

Accepting nerve-tissue as a criterion of the vitality of the constituents of the body, these experiments are very striking, and reveal in a clearer light the profoundly powerful character of chloroform.

In the statistics of deaths under these two anæsthetics ether stands better than the proportion of seven to one. The proportion in its favor is about thirteen to one; but, as Dr. Waller pointed out, the problem of the action of narcotics on the whole organism, although exemplified tersely by a nerve experiment, is in itself far more complex. It is often stated that the after-effects of ether are far more fatal than those due to chloroform; but no foundation in experiment or statistics exists to prove this, and, as Dr. Waller cogently puts it, the use of chloroform, if it be dangerous under all circumstances, is, unless under exceptional circumstances, unjustifiable; and if it is only dangerous when unskilfully given, its deaths amount to homicide. This is Dr. Waller's chloroform dilemma.

The discussion, in which Professor Richet, Dr. Lawrie, Dr. Shore, Professor Gaskell, and Professor Stewart took part, turned mainly upon the oft-debated evidence afforded by cross circulation experiments, as to whether or not the fall of blood-pressure incident to chloroform narcosis is brought about through depression of the vasomotor centre or through weakening of the heart itself. How far such experiments can be accepted as conclusive remains to be seen in the face of so much evidence which has now been accumulated by the researches of MacWilliam, Hare and Thornton, Leonard Hill, and others; it is difficult to exclude the vasomotor centre as in some way causative of the fall of blood-pressure under chloroform. The practical fact which stands out in terrible distinction is that deaths under chloroform have not lessened in spite of physiology or Hyderabad Commissions. It is useless to contend that these deaths arise because the physiologists teach dangerous tenets, since the large percentage of persons who give chloroform know little of and probably care less for physiology. It is rather the careless and the over-confident in whose hands such accidents happen, while, as a rule, it is safer in those of persons who have leisure and training to follow the trend of modern thought and teaching concerning anæsthetics.—*The Therapeutic Gazette.*

